# Medical Department of the Army

**Published:** 1863-10

**Authors:** 


### EDITORIAL DEPARTMENT.

### MEDICAL DEPARTMENT OF THE ARMY.

Medical Inspector Joseph K. Barnes, has been assigned to duty as acting Surgeon-General during the absence of Dr. Hammond, who has been ordered to a distant service, while it is reported that a commission has been appointed to examine into the condition of the Medical Department of the Army, and that pending the investigation the Surgeon-General has been relieved temporarily of duty in the Medical Bureau, as is customary in such cases. Much speculation exists as to the causes of such action, and in this condition of affairs the friends of Dr. Hammond, as well as his opponents, are extreme in their expressions both of favor and opposition.

By the friends of Dr. Hammond it is held that his appointment was calculated to produce, and did excite the envy and jealousy of the senior members of the staff. The practice of selecting the oldest member of the staff for Surgeon-General had become established, and hence it always happened that the duty devolved upon those far advanced in years. While acknowledging that there were senior surgeons who were eminently qualified for the position, it was yet thought best to fully break away from this practice and, Dr. Hammond, an Assistant Surgeon, was appointed to this office, for the following reasons, as his admirers claim: That he was a distinguished medical teacher; in reputation as a medical and scientific author; had experience in military duty; the vigor of middle life; great powers of physical endurance, and *ardent patriotism.*

In regard to the services and labors which the Surgeon-General has bestowed upon the Medical Department of the Army, a great amount of credit is claimed for him. Immense armies, widely scattered, engaged in deadly conflicts, with great numbers of sick and wounded men requiring medical supplies, conveyance, surgical attendance, hospital accommodations, etc., demanded great energy and executive capacity in the chief officer. This duty, it is believed, has been performed with ability, and the character of the medical staff of the army elevated and its efficiency increased. The military hospitals are unsurpassed in their arrangements, and illustrate advanced views in hygienic and sanitary science. If the Surgeon-General has instituted and directed their construction he is entitled to great credit, for they are models in construction and management, and intelligent observers are at once impressed with wonder and admiration. Under the circumstances of construction and management they are certainly a great success, and deserve most unqualified commendation.

Again, reorganization of the Examining Boards, with the view of elevating the standard of qualification for admission, both to the regular and volunteer army; founding the Army Medical Museum for the collection of specimens of military surgery; and the collection and preparation of material for a Medical and Surgical History of the War, designed to illustrate the progress which the profession of the United States has made in Military Surgery, are objects accomplished to such a degree that whoever has been instrumental in proposing and forwarding them, may properly claim both from the profession and public, respectful consideration.

It is also claimed that the organization of an Army Medical School, to be located in Washington, which is so far advanced that the Lecturers are selected for the first course to be given this fall, is another achievement of the Surgeon-General, worthy of great praise. This may be regarded as very praise-worthy by many, but we are not of the number. We have had occasion to change our views upon many subjects connected with military measures and military practice, and possibly the success of this Institution may show us our mistake, but at the present we cannot regard it as either necessary or useful; we look upon it in a very different light. We do not propose to condemn it, or speak disrespectfully of it, but regard it as a scheme for the promotion of personal ambition. If it is so, it may, and probably will be so managed as to advance the knowledge of military surgery in some degree; indeed this is necessary, or the other and more primary object will fail. Perhaps it might as well be stated as a general proposition, applicable in this case as in many others, that medical schools in this country are instituted for two purposes; the first object being the advancement of those who have organized themselves, The Faculty, and agreed to call each other Professors; and the second, the instruction of young men in the primary branches of medicine; this latter object purely incidental, and solely for the promotion of the first grand overshadowing purpose. This scheme of calling each other professors has really become a branch of business, and is now carried to such an extent that when ambitious men cannot by management or merit get others to assist them, it is common to do it alone, and when an aspirant for popular favor takes this course, it is found worth about as much to him as if he had favorite influential colleagues to do him the honor.

Those engaged in this enterprise of professorships are often detected in "laying pipe" upon a plan which will fully equal the lowest tricks of the politician. The present respect which is usually bestowed upon capable and faithful teachers of medical science, will gradually give place for the disgust and contempt which attaches to a cheat, unless some correction of abuses in this direction is obtained. We have no personal objection to the Surgeon-General making a new medical school of his own, and making a class of Professors of Military Surgery. If he does it well, there is nothing to censure, though he should appoint his especial favorites to pleasant positions; but we see, or think we see, appointments made, or to be made, on account of "*ardent patriotism*," through all coming time. Military surgery will be taught by professors of "ardent patriotism" in the Government school, while representatives of distinguished families, or influential circles, or large estates will teach it in the more private colleges. Patriotism which is true, merit which is actual, energy and intelligence which is positive and unconditional, will all be overlooked unless "pipe is laid" for its benefit; *it* will never lay its own "pipe."

Notwithstanding the opposition, which has come up from many quarters, the duties of the office of Surgeon-General have been efficiently and successfully performed, and much of the opposition is the fruit of envy, jealousy and revenge. However, the Secretary has appointed a commission to inquire into the condition and management of the Medical Bureau. What may be the ground for the appointment of this commission does not yet appear, though conjectured rumors of fraud and corruption, are sufficiently numerous.

We have attempted in these remarks to state the general condition of affairs in this department without lengthy comment, or at least to state what appears to us as the truthful condition. The army is an ever fruitful theme, both for the Journalist, the Politician and Divine; all have an interest in its management and success, but it is an especially fruitful source of reflection to the medical profession. It is opening up to it a wide field of labor, and offering opportunity for the cultivation of one important branch of medical knowledge never before studied to any great extent in this country. That there should be great differences of opinion among men, equally well informed, is not strange; that one division of the army surgeons should attach value to that which another equally competent, regard worthless, is not more than is found with intelligent physicians in civil practice, where the field is old, and much better cultivated. Physicians have much to contend with in the practice of their professions, and should learn to bear with the prejudices and peculiarities of others. It is not to be expected that all will think alike of many important therapeutic measures, and the widest range for individual opinion should be given; we should, if possible, become tolerant of each other's opinions. It is pleasant to observe that physicians who attend to their private practice, and have ambition mainly for it, and for a clear perception of truth, have little contention, and comparatively few differences of opinion. Office of whatever description you please, is apt to corrupt and distract all who receive it.—When men accept it, they are to be excused for a great many faults. When it falls upon physicians, the severest judgments have come upon them. We hope, as journalists, to hold upon the exciting medical topics of the army a middle ground, and not too violently censure, or too readily bestow unmerited praise.

